# Improved culture of fastidious *Gemmata* spp. bacteria using marine sponge skeletons

**DOI:** 10.1038/s41598-019-48293-z

**Published:** 2019-08-12

**Authors:** Odilon D. Kaboré, Sylvain Godreuil, Michel Drancourt

**Affiliations:** 1 IHU Méditerranée Infection, Marseille, France; 2 Aix Marseille Univ., IRD, MEPHI, IHU Méditerranée Infection, Marseille, France; 3 Université de Montpellier UMR 1058 UMR MIVEGEC, UMR IRD 224-CNRS Inserm 1058, Montpellier, France

**Keywords:** Bacteria, Applied microbiology

## Abstract

*Gemmata* are Planctomycetes bacteria recalcitrant to traditional cultivation in the clinical microbiology laboratory and they have been seldom documented in patients. Based on previously known relationships of Planctomycetes with marine sponges, we designed a new culture medium A incorporating marine sponge skeleton of *Spongia* sp. to the standard culture medium; and culture medium B incorporating *Spongia* sp. skeleton heat aqueous filtrate into medium A; and inoculating the three culture media (standard, A and B) with *Gemmata obscuriglobus* DSM 5831^T^ and *Gemmata massiliana* DSM 26013^T^ in the presence of negative controls. Cultures were observed by naked eyes for 7 days and bacterial growth was quantified by microscopic observations and culture-based enumerations. Macroscopic observations at day-3 revealed a pink bacterial pellet in medium B tubes while standard medium tubes remained limpid until day-8. Growing *Gemmata* spp. bacteria in medium A yielded air bubbles released by bacterial respiration, whereas control tubes remained bubble-free. The number of colonies in standard medium (1.363 ± 115 for *G*. *obscuriglobus*, 1.288 ± 83 for *G*. *massiliana*) was significantly lower than those counted from medium B (2.552 ± 128 for *G*. *obscuriglobus*, 1.870 ± 112 for *G*. *massiliana*) and from medium A (2.851 ± 137 for *G*. *obscuriglobus*, 2.035 ± 163 for *G*. *massiliana*) (p < 0.10^−4^) at day-2 incubation. At day-3 incubation, the number of colonies counted from supplemented media A and B increased up to one log than those counted from the control medium (p < 0.10^−4^). Along the following day-4–7 incubation, the number of colonies counted from media A and B remained significantly higher compared to standard medium (p < 0.10^−4^). These data indicate that incorporation of spongin-based marine sponge skeleton and heat aqueous filtrate of sponge skeleton significantly improved growth of *Gemmata* spp. bacteria. These observations pave the way towards improved isolation and culture of *Gemmata* spp. from environmental and clinical specimens.

## Introduction

Bacteria of the genus *Gemmata*, phylum Planctomycetes^1^ form a group of organisms of interest in environmental sciences and medicine^2^. In the environment, these organisms have been detected in soil, freshwater and marine habitats^3–7^. However, environmental *Gemmata* spp. form a vast world of mainly uncultured organisms as only *Gemmata obscuriglobus* has been isolated from freshwater^8^ and *Gemmata massiliana* from an hospital water network in close proximity with patients^9^. In the clinical microbiology laboratory also, *Gemmata* spp. organisms are recalcitrant to traditional cultivation, although they have been seldom documented in patients^10,11^. Using a PCR-sequencing approach, we recently found some *Gemmata*-like sequences in the blood collected from two patients with febrile aplasic neutropenia and leukemia, although we failed to isolate any Planctomycetes organism from these patients’ blood specimens in pure culture, despite several attempts^11^. Indeed, *Gemmata* organisms are fastidious bacteria requiring highly specific culture medium^12–14^. Accordingly, conventional automated microbial detection of blood culture system is not appropriate to detect such bacteria (undetected) and less sensitive than culturing mock-infected blood on Caulobacter agar^15^. Nevertheless, their resistance to most of the routinely used antibiotics^16^ and the recently demonstrated association with humans^10,11^ support the potential of *Gemmata* organisms to behave as opportunistic pathogens warranting furthur investigations^2^.

It has been reported that the lack of complex factors/conditions in the laboratory contributed to the inability to isolate some fastidious bacterial species^17,18^. Accordingly, providing environmental and nutritional conditions similar to those existing in the natural habitat where yet uncultured bacteria are detected, may be an option for tentative isolation and culture^17,18^. In marine environments, a large fraction of Plantomycetes reside permanently in sponges pointing to highly integrated symbiotic relationships between the host sponge and Planctomycetes^19^. The reasons for such a symbiosis may be mechanical or nutritional relationships and chemotaxis as nutrients released by marine sponge surfaces may attract bacteria^19^ and provoke their attachment via glycoproteins and uncharacterized holdfast structures. The holdfast of marine bacteria can form temporal or permanent junctions to stabilize the biofilm^20,21^ and may help *Gemmata* organisms to ensure a rapid growth and their reproduction by budding^8,22,23^.

Based on these knowledges, we hypothesized that creating in the laboratory an attached-living style using dead marine sponge tissues (spongin) may act as a growth-promoting condition to improve the culturabily of *Gemmata* organisms. We sought to test the growth-enhancement effect of complementing *Gemmata* species standard culture medium with marine sponge filtrate and sponge small fractions as a solid phase to mimic planctomycetes natural environment in order to develop a new biphasic culture system for *Gemmata* spp. bacteria.

## Results

### Organoleptic characteristics of the liquid media and macroscopic-microscopic structures of dead sponge skeleton

By observing the organoleptic traits of the three different media after autoclaving, we observed a more yellow color of the media supplemented with marine sponge extracts while the unsupplemented Caulobacter standard liquid medium remained very light straw yellow. It is probable that the yellow color resulted from the diffusion of chemical compounds from sponge after autoclaving since the sponge, heated in deionized water alone (without Caulobacter components) showed a slight yellow color. However, the autoclaving process did not significantly alter the macroscopic and microscopic structures of the sponge skeleton compared to control unautoclaved sponge skeletons.

### Macroscopic observations

In stationary broth cultures, the macroscopic appearance of the supplemented broth changed considerably during the course of culture. Indeed, growth occured in the form of a pink pellet that developed at the bottom of the tube. The pink pellet appeared at day-3 (*G*. *obscuriglobu*s) and day-4 (*G*. *massiliana*) in the medium B, while it appeared at day 7–8 for *G*. *obscuriglobus* and at day 10–11 for *G*. *massiliana* in control medium. In medium A growth occured with proportional increase of the air bubbles in the medium but the pink pellet did not appear in parallel in medium B. Indeed, for medium A, at day-1 post-inoculation, we observed air bubbles that increased significantly in number at days 3–4 and throughout the experiment up to a peak at day 6–7 for inoculated media while the tubes containing non-inoculated medium A exhibited no air bubbles. Also, after being vortexed, the enriched media showed a stable foam of at least 2.5 cm above the liquid (inoculated and uninoculated) for 5 to 7 min while a small layer of the foam of 2 mm disappeared after 30 seconds in the unsupplemented medium (inoculated and non-inoculated tubes). At day-14 post-inoculation, this foam did not appear after the vortex process for inoculated supplemented media while it always appeared from non-inoculated medium. These observations suggested the possible presence of amphiphilic molecules from the sponge skeleton medium that have been dissolved in the standard medium. Also, the fact that the foam that appeared at the beginning of the experiment did not appear at day-14 post-inoculation suggested that some molecules, which formed this foam, were completely consumed by the bacteria or bacterial growth has induced an inhibition of this foam, due to the releasing of acid toxic products pH (3.5–4) in the medium. By vortexing the tubes, the supplemented media appeared to be in well-clouded at the 3 rd day of the experiment compared to the non-supplemented media that remained limpid until the small pink layer began to appear at the day 7–8 post-inoculation (*G*. *obscuriglobus*) and day 10 post-inoculation (*G*. *massiliana*).

### Microscopic observations

Under our microscopic visualization, all non-inoculated, negative control tubes remained sterile over the entire experiments, and culture on soild media provided confirmation. The composition of the medium was an important factor affecting both the rosettes formation (*G*. *massiliana*) and the latent period before the apparition of budding cells (*G*. *massiliana* and *G*. *obscuriglobus*). In inoculated tubes, both strains were ovoid with a pleomorphism characteristic of bacteria from supplemented broth and usually occurred in pairs (mother cell with small bud located at one pole) *versus* singly from unsupplemented medium at day-1 post-inoculation. Indeed, at one day post-inocalation, in the medium supplemented with small fractions of sponge as solid support, 40 ± 7% of *G*. *massiliana* cells appeared to possess one daughter cell (small bud) located near the mother cell and 46 ± 13% of *G*. *obscuriglobus* bacteria possessed daughter cell located near the mother cell. Also, budding bacteria counted from the medium containing sponge filtrate alone represented at one day post-inoculation, 38 ± 12% for *G*. *massiliana* and 43 ± 11% for *G*. *obscuriglobus* while bacteria visualized from the control media showed a lower proportion of 10 ± 4% for *G*. *massiliana versus* 15 ± 6% for *G*. *obscuriglobus* bacteria with one bud located at a pole of mother cell. On solid agar, these small buds after their growth can be seen near one of the two poles of the mother cell (which appears bigger). Without the small fractions of sponge as solid support in the medium supplemented with marine sponge filtrate and the standard Caulobacter medium, the pellet resuspended contained small and large rosettes (8–20 cells per rosette for sponge filtrate medium and 4–12 cells per rosette for control medium compared to medium containing sponge fractions (P < 0.001) where the cells appeared separated, attached to sponge skeletons, singly, pairs, pleiomorphic or forming small rosettes (4 to 6 cells). These observations suggest that in the absence of sponge as a solid support, bacteria tend to form large rosettes and thus constituting their own “self-support” to ensure their budding. In contrast, with the presence of sponge as a solid support, bacteria tend to cling, preferably, to the sponge fragments to ensure their budding.

### Colonies-based microbial enumeration on Caulobacter solid medium

**Figure 1:**
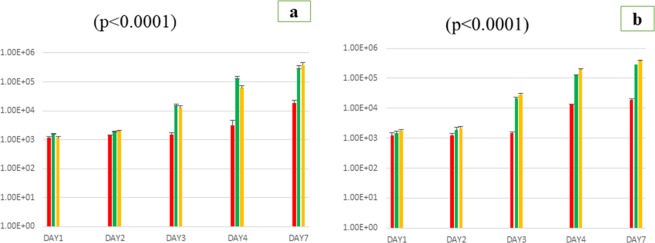
Growth-enhancement effect of complementing *Gemmata obscuriglobus* standard culture medium (red bar) with marine sponge filtrate (green bar) and sponge small fractions as a solid phase (yellow bar). Bacteria were counted on Caulobacter solid agar plate after 1 × 10 s (**a**) and 4 × 10 s (**b**) of vortex process. The number of *G*. *obscuriglobus* colonies (Y axis) was monitored along a 7 days period (X axis). Data presented are for a 7 days period culture. Each data point represents the mean ± SD of five experiments. Standard errors are represented by error bars.

The negative control plates remained sterile over the entire experiments. In the media containing sponge extracts, growth yield was not proportional to the sponge extract concentration from 0.5 g to 1 g. At sponge extract concentrations of 0.5% (M/V), the viscosity remained roughly similar compared to the standard medium and did not compromise the bacterial enumeration as standard deviation values were optimum in the medium containing sponge fractions (preliminary studies, data not shown). *Gemmata obscuriglobus* colonies sub-cultured in each broth of the three tested liquid media, were seeded at each time (as described in the method section) on Caulobacter solid agar and then counted after a 14-day incubation period at 30 °C. Thus, colonies enumerations revealed that the cultures of *G*. *obscuriglobus* from both supplemented broth, *i*.*e* sponge filtrate broth, and sponge small fractions immersed in sponge filtrate broth (biphasic system) were significantly higher than cultures obtained with Caulobacter standard broth (p < 0.0001). Indeed, from standard Caulobacter broth, the number of *G*. *obscuriglobus* colonies counted was (1.162 ± 51) at day-1, (1.363 ± 115) at day-2, (2.390 ± 427) at day-3, and (6.800 ± 810), (270.000 ± 2.100) at day-4 and day-7 post-inoculation respectively. Supplementation of the standard Caulobacter broth with sponge filtrate increased significantly the number of *Gemmata obscuriglobus* colonies (p < 0.0001) and the colonies counted after seeding onto solid agar plates were (1.792 ± 86 colonies) at day-1, (2.552 ± 128) at day-2, (17.000 ± 1.120) at day-3, (130.000 ± 1.270), (2.775.000 ± 35.000) at day-4 and 7 post-inoculation respectively. Likewise, the addition of sponge small fractions in the standard broth supplemented with sponge filtrate (biphasic system) showed that the number of *Gemmata obscuriglobus* colonies was (1.146 ± 66) at day-1, (2.851 ± 137) at day-2, (19.750 ± 1.300) at day-3, and (279.600 ± 1.277), (3.220.000 ± 46.000) at day-4 and 7 post-inoculation, respectively (Fig. 1a). In parallel to colonies enumerations from agar plates, microscopic monitoring showed the rosette of cells and attached bacteria to sponge despite the 10 secondes vortex process. We had then increased the vortex process until the low standard deviations values (described in method section) has been obtained (4 × 10 s). Thus, the following enumerations after the serial vortex resulted to the very high increasing of colonies from the medium supplemented with small fractions of marine sponge (biphasic system), as shown in (Fig. 1b), compared to the two other media (monophasic systems).

**Figure 2:**
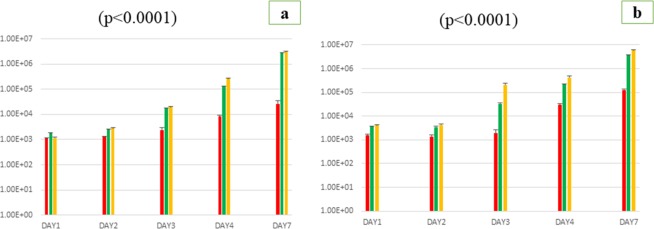
Growth-enhancement effect of complementing *Gemmata massiliana* standard culture medium (red bar) with marine sponge filtrate (green bar) and sponge small fractions as a solid phase (yellow bar). Bacteria were counted on Caulobacter solid agar plate after 1 × 10 s (**a**) and 4 × 10 s (**b**) of vortex process. The number of *G*. *obscuriglobus* colonies (Y axis) was monitored along a 7 days period (X axis). Data presented are for a 7 days period culture. Each data point represents the mean ± SD of five experiments. Standard errors are represented by error bars.

Likewise, the same effects of the addition of sponge extracts were observed with *Gemmata massiliana*. Indeed, bacteria sub-cultured in each broth of the three tested liquid media were plated on Caulobacter solid agar and then counted after a 21-day incubation period. Thus, the number of *G*. *massiliana* colonies enumarated from Caulobacter standard medium was (1.193 ± 96 colonies) at day-1, (1.288 ± 83) at day-2, (1.480 ± 122) at day-3, and (4.100 ± 314), (28.000 ± 2.550) at day-4 and day-7 post-inoculation respectively, whereas the number of colonies counted from medium supplemented with sponge filtrate was (1.527 ± 89 colonies) at day-1, (1.870 ± 128) at day-2, (11.000 ± 2.100) at day-3, and (62.000 ± 2.260), (220.000 ± 4.200) at day-4 and 7 post-inoculation respectively. The addition of sponge small fractions for bacterial attachment in the standard broth supplemented with sponge filtrate (biphasic system) increased the growth rate and colonies enumerations were (1.166 ± 72 colonies) at day-1, (2.035 ± 128) at day-2, (12.600 ± 2.300) at day-3, and (93.000 ± 3.900), (280.000 ± 5.600) at day-4 and day-7 post-inoculation respectively (Fig. 2a). As described for *G*. *obscuriglobus*, the enumeration of *G*. *massiliana* colonies after the serial vortex resulted in a very high increasing of colonies from the biphasic medium supplemented with small fractions of marine sponges than the two other monophasic media, as show the (Fig. 2b). From day-7 to day-14 post-inoculation, the depletion of nutrients and potential growth factors thus marks the stationary phase of bacterial growth while the bacterial growth progressed in the unsupplemented medium.

### Growth on solid media

On the surface of both media, both colonies were circular, convex and had entire margins with sometime a litle bud (daugher cell) near to mother cell. *G*. *massiliana* organisms formed colorless colonies (pink), while *G*. *obscuriglobus* colonies were pink-red. Relatively, the growth of both organisms from medium supplemented with marine sponge filtrate was more rapid, with small colonies becoming visible at day 5 (*G*. *obscuriglobus*) and days 7 (*G*. *massiliana*) *versus* day 8 (*G*. *obscuriglobus*) to day 11 (*G*. *massiliana*) in unsupplemend solid medium at 30 °C (size < 0.5 mm). The maximal colony diameter varies somewhat with the strain, but usually reaches 1 to 3 mm after 14-day (*G*. *obscuriglobus*), 21-day (*G*. *massiliana*) incubation from medium supplemented with sponge extract *versus* 1 to 2 mm diameters from unsupplemented solid medium.

## Discussion

Although *Gemmata* spp. sequences have been detected in stool specimens^10^ and in the blood of two patients with febrile neutropenia^11^, no isolate issued from tentative culture of these clinical specimens. Failure to isolate any *Gemmata* organism from these specimens may rely on the fact that PCR-based detected organisms were dead and indeed, no characterization of their viability has been attempted. Alternatively, failure to isolate and culture may be due to inappropriate culture conditions of the clinical specimens. Blood culture remains the gold standard for diagnosing bloodstream infections but conventional cultivation of *Gemmata* microorganisms from blood culture is requiring specific conditions for blood collection and culture^15^.

Mimicking culture strategies by environmental microbiologists, we aimed to incorporate marine sponge skeleton fragments and spongin-based sponge skeleton extract in the culture medium routinely used to grow *G*. *obscuriglobus* and *G*. *massiliana* organisms, because of some other Planctomycetes have been shown to have tight relationships with marine sponges^19,24,25^. Indeed, our observations revealed a significant growth-promoting effect after complementing Caulobacter standard medium with marine sponge skeleton filtrate. These results imply that some yet unknown thermosoluble growth factors are strongly and possibly bound to the sponge skeletons and have been dissolved by heat (autoclaving) in supplemented media. The existence of an intimate relationship between planctomycetes and marine sponges^19^ can be ascribed (partly) to these observations via a mounting molecular evidence, stimulating the rapid growth of slow-growing bacteria like planctomycetes.

*Spongia* sp.(Demospongiae: Porifera)^24^ consist of sponges whose skeleton is mostly made up of a composite of natural biomaterials containing organic constituents like protein spongin (a network of organic collagenous analogous to collagen type XIII), polysaccharides and/or inorganic compounds, which may have been incorporated into the spongin structure from the environment^25–29^. Spongin consists mostly of carbon, nitrogen, oxygen and hydrogen and the the presence of sulfuris connected with the disulfide bonds of cysteine has also been reported in spongin structure^30^. Sponginous collagens analysis of the sponge *Spongia officinalis obliqua* revealed that not only proteinogenic amino acids but also halogenated (Brominated) tyrosines were occured in these sponges^31^. More reports have pointed that sponge collagen is a good biomaterial for medical^32^, pharmaceutical^33^, nutraceutical^34^ and cosmeceutical^34–36^ applications (bone tissue regeneration, moisturizer in cosmetic formulations). Indeed, collagen has the properties related to its gel formation, surface behavior, which includes emulsion, foam formation, stabilization (stable foam occurred in our tested media, see above for details), adhesion, protective colloid function and film-forming capacity. In addition, collagen is a good surface-active agent and has an ability to penetrate a lipid-free interface^37,38^. All these propreties make it a good component which are likely contributed to improve the *Gemmata* nutrition, which are bacteria well-known to uptake of such macromolecules by their endocytosis-like process^39,40^.

Additionnaly, some of sponge skeletons solubilized molecules by heat in our enriched media are likely Glycosaminoglycans (a source of N-acetyl-glucosamine, N-acetyl-galactosamine, uronic acid), a well-known polysaccharide from sponginous skeletons of *Spongia officinalis* and *S*. *lamella* with a total content of 0.367 ± 0.028 and 0.460 ± 0.081 (µg hexuronate/mg dry weight), respectively^31,35^. The N-acetyl-glucosamine from these Glycosaminoglycans represents a good source of both carbone and nitrogen for planctomycetes nutrition^12,13^ and this can explain (partly) the molecular evidence stimulating the rapid growth of slow-growing *Gemmata* spp. Hence, sponge skeleton filtrates are a good source of several well-known molecules but, it seems that spongin chemistry is very complex by the presence of many yet unknown molecules which have never been reported and which have certainly contributed to enrich again our supplemented media.

On the other hand, we observed that adding marine sponge small fractions (medium A) to culture of *Gemmata* spp. bacteria resulted in highly significant increases in bacterial growth (*P* < 0.0001), compared to controls. We interpreted these observations as indicative of a mechanical effect of promoting growth. In the nature, the planctomycetes (genus *Rhodopirellula*) exhibit an attached life style to Mediterranean sponge *Aplysina aerophoba*^14,19^. The presence of a holdfast of glycoproteic nature favors attachment and, thus, the colonization of surfaces^41^. The marine sponge *Spongia* sp. skeleton resemble in its organization and composition the connective tissue of vertebrates in that it is composed of collagen fibers and fibrils embedded in an amorphous matrix containing carbohydrates^26^, which support the adhesion and have already proven to promote growth and differentiation of human mesenchymal stem cells into osteoblast cells^42–44^. It is the mechanical and material properties of the skeleton of these sponges which would favor *Gemmata* holdfasts attachment and likely could activate a signal transduction through a second messenger to accelerate the budding, which would result of an increasing cell division.

In summary, the very rapid growth kinetics observed from supplemented media may be due to some well-known (N-acetyl-d-glucosamine) and/or unknown soluble chemicals released by hot water from the sponge skeleton tissues for *Gemmata* metabolism. The sponge may also have promoted mecanically the rapid growth by providing a microenvironment for bacterial attachment and an increasing of bacterial division. Despite the 2–3 days gained after the enriched solid media by the sponge skeleton filtrate, the growth of these fastidious bacteria remains slow on this enriched solid medium. Further works should be undertaken to lyophilize these sponge skeleton filtrates or by grinding fresh sponge tissues to enrich fastidious germs culture media. A culture in a sponge skeleton liquid medium is necessary as enriched medium before plating on sponge filtrate solid agar for isolation.

In conclusion, our study provids here, a very promising technique which could be easily implemented in microbiology laboratories. Although scientists can enrich slow-growing microorganisms using several methods as diffusion chambers^45^ or soil substrate membranes^46^, most enriched bacteria (uncultured *Gemmata*-likes) could not grow on agar plates for isolation and further cultivation because, in fact, during the enrichment step, fast-growing microorganisms may overcome slow growing bacteria, leading to reduced diversity of bacterial species. This technique may help confirm the involvement of *Gemmata*-like microorganisms in infectious processes in aplastic patients or in pulmonary disease in the intensive care unit, which are not documented microbiologically yet.

## Materials and Methods

### Bacterial strains and culture conditions

*G*. *obscuriglobus* DSM 5831^T^ and *G*. *massiliana* DSM 26013^T^ (CSUR P189^T^) were obtained from the German Collection of Microorganisms and Cell Cultures (Braunschweig, Germany) and the Collection de Souches de l’Unité des Rickettsies (Marseille, France), respectively. Bacteria were sub-cultured on solid Caulobacter medium DSMZ 595 and in liquid Caulobacter medium DSMZ 595, prepared as described on the website (http://www.dsmz.de). These two-culture media were used as standard control media in next experiments. Bacteria were grown on these media incubated aerobically at 30 °C for 7 to 14 days. Identification of colonies was then ensured by matrix-assisted laser desorption/ionization time-of-flight mass spectrometry (MALDI-TOF-MS) analysis as previously described^31^.

### Marine sponges

**Figure 3:**
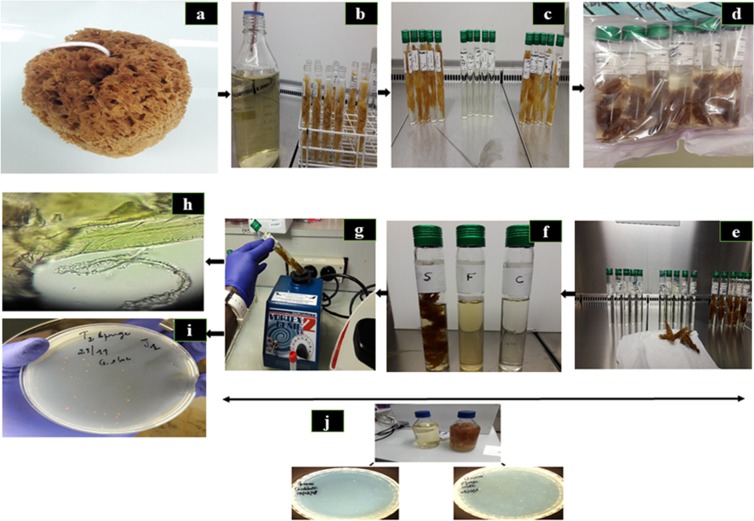
The main steps of experimental procedure: (**a**) Dead *Spongia* Sp. skeleton were washed and dried. (**b**) Sponges were cut into small pieces of uniform size and 0.5 g were introduced in glass tubes before adding 15 mL Caulobacter liquid medium (CLM). (**c**) The three-liquid media before autoclaving: ten glass tubes containing 0.5 g small fractions of sponges immersed in 15 mL CLM (medium B), and five glass tubes containing 15 mL. (**d**) Marine sponges immersed in the CLM after autoclaving at 121 °C/15 min **e**. The three media after autoclaving: at left to right: control medium, medium B (prepared as described for medium A except that the 0.5 g small pieces of sponge tissue have been removed after autoclaving and filtered and medium A (a liquid–solid biphasic system). The final volume of culture at 15 mL was then completed after an adjustment with each corresponding liquid medium after autoclaving in order to compensate the liquid evaporated. (**f**) The characteristics of the three liquid media prepared to be inoculated. (S) for sponge medium, (F) for filtrate and (C) for CLM. (**g**) Procedure of vortex. Each tube was vortexed for 1 × 10 s and broth was removed to perform serial dilutions followed by subculture onto agar solidified medium on 100-mm Petri dishes. Vortex procedure was repeated (4 × 10 s) until a constant enumeration was obtained. Bacterial growth was monitored by microscopy (using kova slides) (**h**) and CFUs enumeration (**i**) in parallel. (**j**) Culture on solid media. For control medium and sponge filtrate medium, 15 g/L of agar were added to prepare corresponding solid media: sponge filtrate (**F**) solid medium, and (C) Caulobacter solid medium.

Natural dead sponges skeleton *Spongia* sp. (Fig. 3a) were purchased from three local suppliers in Marseille, France (local suppliers are selling natural art objects including unbleached marine sponges and other products) at different times for successive experiments. Researchers should be careful, the color of the sponge must be dark yellow to maroon color and unscented, compared to skeletons of natural sponges totally bleached and sold in soap shops and pharmacies (very white and purified, personal comparison). Sponges were soaked in 9.6% bleach (sodium hypochlorite, Laboratoire Oxena, Romans-sur-Isère, France) solution for 3 minutes and then washed in deionized water as described elswhere^47^. Sponges were then rinsed three times by immersion in deionized sterile water for one hour and then dried at room temperature. Afterwards, sponges were cut into 0.5 g pieces (Fig. 3b).

### Experimental procedures

All experiments incorporated five replicates, and were repeated five times for ensuring reproducibility, and were done independently with each one of the two cultured strains.

### Experiments on liquid media

Three liquid media were prepared: (i) five glass tubes (Bio-Rad Laboratories, Hercules, California) containing 15 mL Caulobacter liquid medium as control medium (ii) five glass tubes containing 15 mL Caulobacter liquid medium plus accurately weighed 0.5-g fractions (in order to increase the surface of bacterial attachment) of marine sponges, immersed in the Caulobacter liquid medium, a liquid–solid biphasic medium refered as medium A (iii) five glass tubes containing 15 mL of medium A except that the 0.5-g pieces of sponge tissues were removed after autoclaving and filtered to yield a liquid monophasic medium refered as medium B (Fig. 3c,d,e) (see Fig. 3). All media were sterilized by autoclaving at 121 °C for 15 min. and the pH was adjusted to 6.8 by the dropwise addition of KOH (10M). Also, the viscosity of the media was grossly assessed by depositing a drop on a solid agar, inclined at an angle of about 30 degree and allowing the liquid to flow. In each experiment, batches comprised of five inoculated tubes and three negative controls, non-inoculated tubes. Prior to inoculation, *Gemmata* spp. bacteria were suspended in sterile water, calibrated at 10^3^ bacteria/mL using a Kovas slide 10 (Hycor biomedical, Germany) and 10 µL of this suspension were inoculated into each one of 5 tubes containing control Caulobacter medium, 5 tubes containing medium A and 5 tubes containing medium B (Fig. 3f). In parallel, 10 µL of sterile water were added in the 3 negative control tubes. The preparations were then incubated at 30 °C in an aerobic atmosphere for seven days. Bacteria growth was examined daily by microscopic observations and culture-based enumerations of the Colony-Forming Units (CFUs), from day 1 to day 7. At each time-point, each tube was vortexed for 10 seconds (Fig. 3g) and broth was removed to perform microscopic observations (Fig. 3h) and serial dilutions of 1, 1/10, 1/100, 1/1.000, 1/10.000 followed by subculture onto Caulobacter solidified medium on Petri dishes (Greiner, Frickenhausen, Germany) (Fig. 3i). In order to unhook and to disperse bacteria, vortexing was repeated for 4 times until a constant enumeration was obtained (low standard deviation values). Only the results of the first vortexing (1 × 10 s) and 4th vortexing (3 × 10 s) were presented. Bacteria were then counted after two-week incubation at 30 °C under aerobic atmosphere. Optical microscopic observations were carried-out on the sponge tissues before and after autoclaving. Also, microscopic observations were performed in the presence or without trypan blue staining at fresh state using Kova slide, in order to monitor bacterial attachment to marine sponges’ small fractions, bacterial abundance, rosettes formation and their morphology from each medium.

### Experiments on solid media

Experiments were also conducted to evaluate the time of growth and the colonies features on solid media (distilled water containing 0.2% peptone, 0.1% yeast extract, 0.02% MgSO_4_1S 7H20 and 1.5% agar). Indeed, for control medium and medium A described above, 100-mm Petri dishes containing 15 g/L of solid agar were prepared to contain each component reported above (Fig. 3j). For medium A, 500 µL of marine sponge filtrate were added on the plate and dried for 30 minutes in a laminar flow cabinet and 500 µL of Caulobacter liquid medium were added in the control Petri dishes. Non-inoculated, negative control Petri dishes were manipulated in parallel. Agar plates were incubated at 30 °C for 2–3 weeks under aerobic conditions and monitored every day. *G*. *massiliana* and *G*. *obscuriglobus* were cultured independently in the same manner.

### Statistical analysis

Means and standard deviation values were calculated at each time (five replicates, n = 5). All the experiments were reproduced independently in the same manner with *G*. *obscuriglobus* in parallel to *G*. *massiliana*. Results are expressed as CFU per mL. Statistical analysis one line (http://marne.u707.jussieu.fr/biostatgv/) was used to conduct an analysis of variance and least significant difference mean separation tests (P < 0.05).
